# Complications following Tension-Free Vaginal Tapes: Accurate Diagnosis and Complications Management

**DOI:** 10.1155/2015/538391

**Published:** 2015-04-20

**Authors:** J. Kociszewski, S. Kolben, D. Barski, V. Viereck, E. Barcz

**Affiliations:** ^1^Department of Gynecology and Obstetrics, Lutheran Hospital Hagen-Haspe, Brusebrinkstraße 20, 58135 Hagen, Germany; ^2^Department of Urology, Lukas Hospital, Preussenstraße 84, 41464 Neuss, Germany; ^3^Department of Gynecology and Obstetrics, Cantonal Hospital, Pfaffenholzstraße 4, 8501 Frauenfeld, Switzerland; ^4^Department of Obstetrics and Gynecology, Medical University of Warsaw, Żwirki i Wigury 61, 02-091 Warsaw, Poland

## Abstract

The sling procedures are the gold standard for SUI treatment. They are highly effective but not free from complications. The most common adverse effect for the surgery with the implant insertion is: overactive bladder occurring de novo after the surgery, voiding dysfunctions, urine retention, and unsatisfactory treatment outcome. The most important question that arises after 20 years of sling procedures is how to manage the complications and what can be offered to complicated patients. The above review summarises the ultrasound findings in complicated cases and shows the scheme of management of the clinical problems concerning the tape location in suburethral region.

## 1. Introduction

Ultrasound examination of the lower urinary tract has been established within urogynaecology as a simple, prompt, reproducible, and dynamic diagnostic procedure [1–5]. Compared to other methods, ultrasound imaging provides more accurate and perfect visualisation of both anatomy of lower urinary tract and dynamic changes in bladder neck mobility during functional tests (Valsalva manoeuvre, cough test) as well as visualisation of synthetic implants. Without radiation exposure, it offers images that are comparable with X-ray methods [6, 7]. Compared with the time and cost consuming functional MRI, ultrasound examination offers advantages because of the simple demonstration in previously mentioned areas. Moreover, the costs and learning curve are the arguments for choosing this method for the first line diagnosis in urogynaecology [8].

Nowadays ultrasound has become one of the most essential diagnostic methods in urogynaecology [4]. Perineal and introitus ultrasonography are two standardised methods, which have been available for years and are already well established [4, 5].

The above methods differ from each other by transducer and probe placement.

The linear or curved array probe is used in perineal sonography and is placed on the perineal and vaginal area [4, 7]. The advantage of perineal ultrasound examination is the short learning curve. With low ultrasound frequency and a large angle of reflection it provides a wide view of the pelvis. Without a doubt, this device setup allows a view that at the beginning often confirms the initial preliminary diagnosis. The large angle of reflection is however coherent with a lower image frequency. This can be problematic when addressing specific issues such as transient, short-term funneling of the urethra.

In the introitus ultrasound examination used for diagnostic of bladder function, a vaginal transducer is positioned in the introitus area over the meatus urethrae externus, thereby ensuring that the direction of the probe axis is strictly orthograde to the patient's body axis imaging performed in both the resting and contraction of the pelvic muscles. The probe should not be inserted into the vagina in order to avoid artificial dislocation of the cystourethral region. This orthograde positioning of the ultrasound transducer is crucial for correct location of the bladder neck (level reading H and distance D), descensus type of the urethrae, and lower base of the bladder/bladder floor (vertical descensus of the urethrae, rotatory descensus of the urethrae, mixed forms of the urethra descensus, and central cystocele). The introitus ultrasonography positions itself between vaginal and perineal one [9]. Further advantages of introitus ultrasonography include the improved resolution of the high frequent vaginal transducer and the possibility to carry out an urodynamic measurement during the same examination.

Both ultrasonographic procedures are standardised and deliver reproducible results. They were developed for the anatomical analysis of the urethra-bladder region within the framework of advanced urinary incontinence diagnosis [3, 7, 9].

The urogynaecological examination must include the separate analysis of all compartments including both the incontinence and the possible incidence of pelvic organ prolapse in order to confirm a clinical suspected diagnosis. When the standard perineal and standard introitus ultrasonography is used, the anterior compartment could be clarified.

However, as we know from our daily practice, the anatomical defect of one compartment can positively or negatively influence the function of another parameter; for example, kinking of the urethra, large cystocele, or a rectoenterocele may lead to a voiding disorder, to an overflow incontinence or masked stress urinary incontinence.

The individual compartments of the pelvis must be objectively visualised with an imaging method in order to better understand the pathomorphological abnormalities of the pelvic organs and to achieve the optimal treatment approach. Furthermore the pelvic floor ultrasound examination can lead to a new ultrasound concept, whereby the introitus/vaginal/endoanal and abdominal ultrasonography in both 2D and 3D techniques can be combined in one investigation procedure [10].

In contrast with the already established standard ultrasonography techniques such as perineal and introitus ultrasonography, the two-dimensional pelvic floor ultrasound examination enables a real-time, static, and dynamic imaging with easy transition of the pelvic compartments in three views: sagittal, frontal, and axial planes.

Compared with the standard ultrasonography, the vaginal transducer probe can be used for both vaginal and consecutive introitus ultrasonography, delivering a new dimension of diagnostic possibilities.

Moreover the PF (pelvic floor) ultrasonography offers the adequate conditions to monitor the position and function of the tension-free vaginal sling [11–15].

## 2. Implants Visualisation in Pelvic Floor Ultrasound Examination and the Proposal of Complications Management

Four parameters can be used to evaluate a tape position.

*(A) Sagittal Plane*

1. *The position (L)* of the sling is in relation to the length of the urethra (at rest).
2. The optimal position of the tape is the distal one-third of the urethral length for TVT-procedure in the middle part of the urethra length for TOT-procedure [16].
3. *The distance (A)* of the sling to the LSM complex (longitudinal smooth muscle complex) of the urethra is optimal between 3 and 5 mm [16].
4. *The shape (F)* of the sling: parallel to the urethra, smoothly stretched, without the horseshoe shaped bending. During Valsalva maneuver a bending indicates a usage of the elastic reserve of the implant. The above confirms a good “tape functionality.”

*(B) Frontal or Axial Plane*

(4) *The symmetry (S)* of the sling: no lateral contact or compression of the urethra.

**Figure 1:**
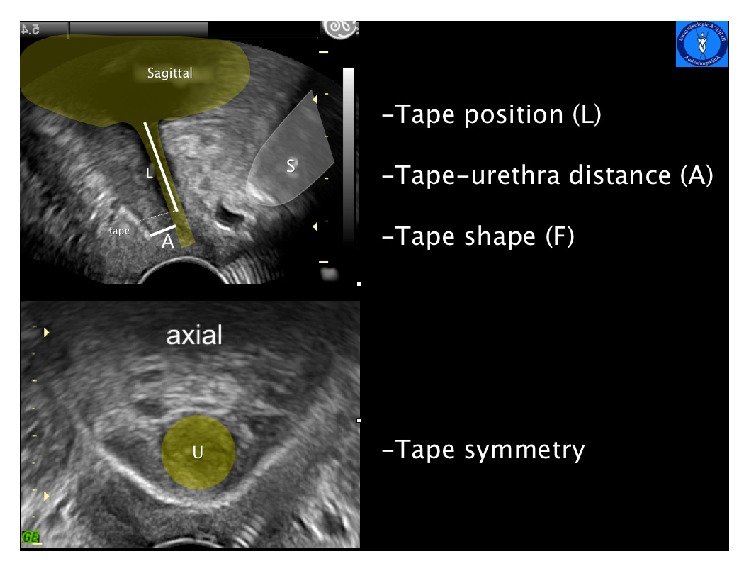
Pelvic floor-ultrasound images in a sagittal plane (above) and axial plane (below). S: symphysis pubis, U: urethra, L: tape position in relation to the urethra length, and A: shortest distance of the tape from the LSM complex of the urethra.

Criteria to evaluate a tension-free vaginal sling and orthotropic tape position are presented in Figure 1.

We consider it particularly important to evaluate the sling position in the first postoperative days. Between the first and seventh postoperative day (*early complications*) it is possible to do the necessary corrections and in most cases it is possible to preserve the sling. Addressing a failed position of the sling at a later stage (*late complications*) results in the removal of the implant and after a successful healing period it is possible to reinsert a new sling.

### 2.1. Early Complications (<7 Days)

The most common complications are voiding disorders or urge complaints [17–20]. The pivotal question is whether the problem results from a failed position of the implant or if there is another cause of the problem. The most common clinical presentation is cystitis, postoperative swelling of the tissues, a hematoma, or incorrect micturition.

The ultrasonography evaluation of a well-positioned sling provides certainty that a success of conservative therapy can be expected.

In case of a dystopic position of the implant the first step is to evaluate the sling location and to decide whether or not the band can be saved.

For teaching purposes the urethra can be halved and a dystopic sling position is divided into two groups.

**Figure 2:**
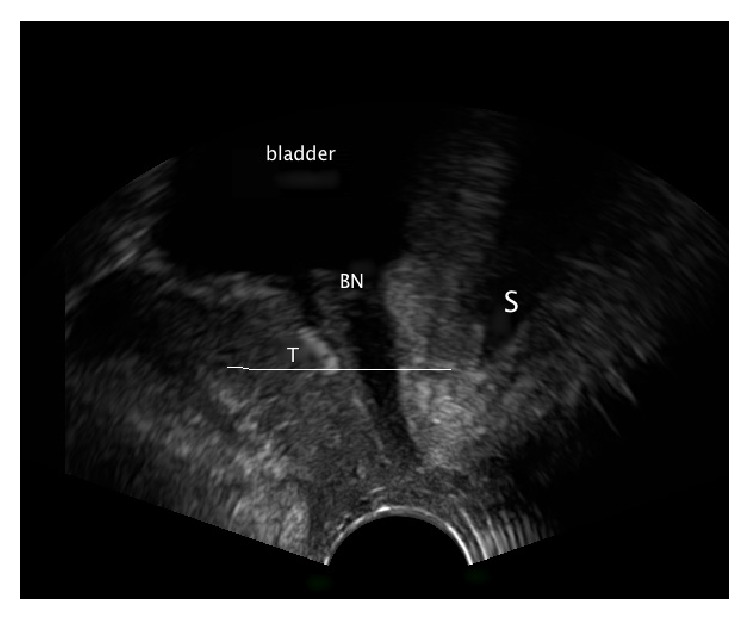
Pelvic floor ultrasound examination in sagittal plane. S: symphysis pubis and BN: bladder neck. The sling lies above the middle part of the urethra.

- A high position of the implant: the middle of the sling (at rest) lies in the proximal half of the urethra. In this case there is no possibility to preserve the implant as the change of its location is impossible (Figure 2).

**Figure 3:**
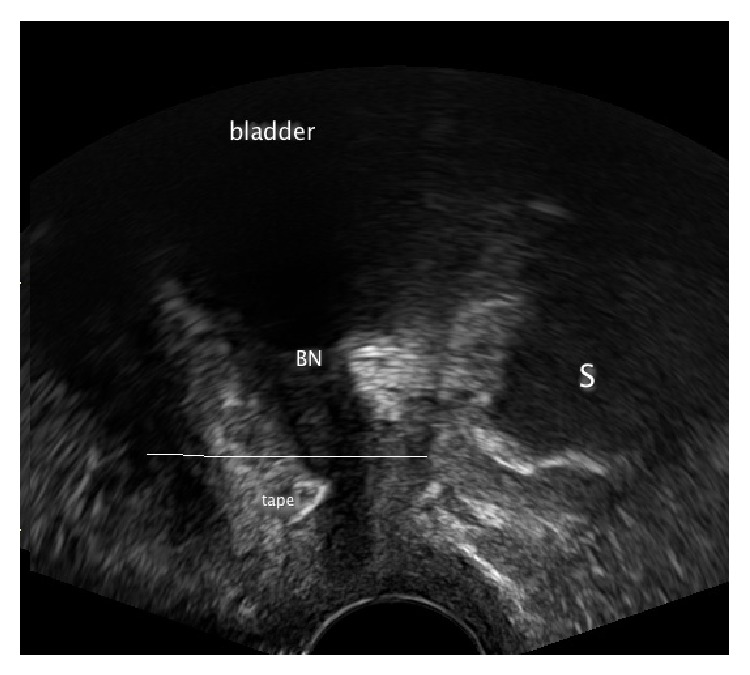
Pelvic floor ultrasound examination in sagittal plane. S: symphysis pubis and BN: bladder neck. The sling lies below the middle part of the urethra.

As it was mentioned above the implant placed in high failed position cannot be corrected; therefore it should be removed and following a healing phase, a new sling insertion may be planned and carried out.

(ii) A low position of the sling: the middle of the implant lies in the distal part of the urethra. In this case there is a possibility of sling preservation (Figure 3).

In the case of a* low faulty position*, for example, a too narrow sling position, lateral compression of the urethra, tethered tape, or dystopic position resulting from a hematoma-early correction of the sling position is usually successful.

**Figure 4:**
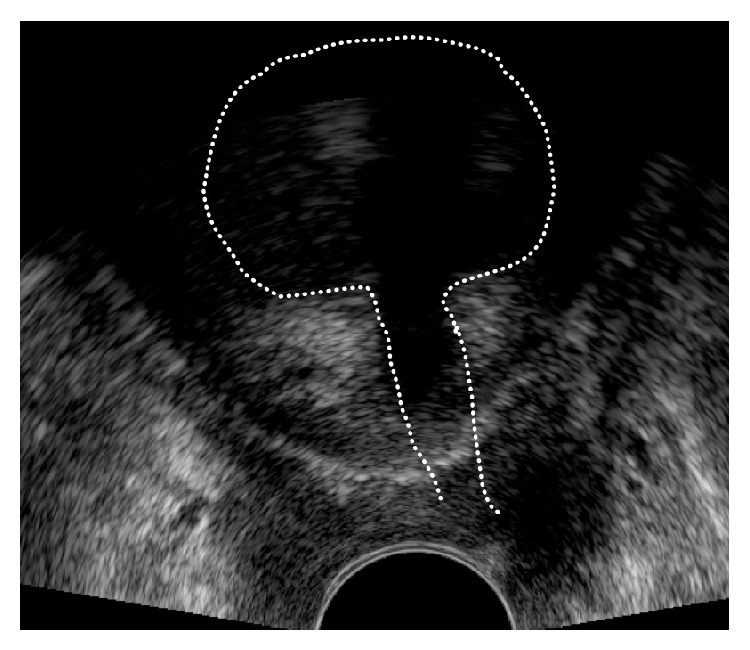
Pelvic floor ultrasound examination in frontal plane. Asymmetric sling position. On the right hand side of the picture the distance of the sling to the LSM complex is much shorter than on the left hand side. A loosening of the implant on the right hand side should be undertaken.

**Figure 5:**
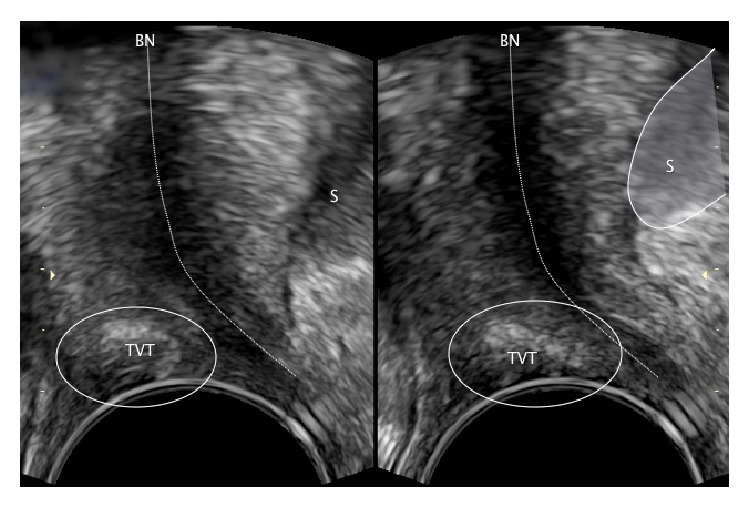
Pelvic floor ultrasound examination in sagittal plane. Shape changes of the sling with a vaginal probe, a typical pathognomonic ultrasound sign for tethered tape. S: symphysis pubis, BH: bladder neck, and TVT: band.

**Figure 6:**
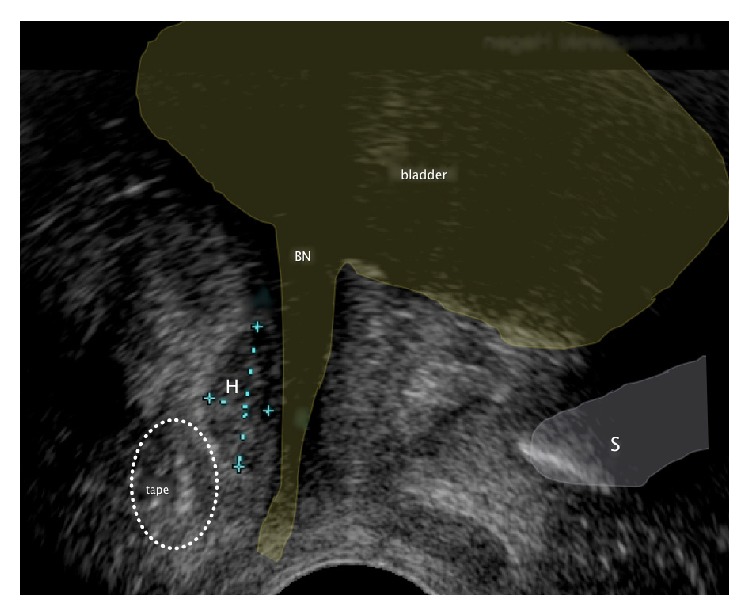
Pelvic floor ultrasound examination in sagittal plane. A small hematoma between TVT and the urethra leads to compression of the urethra and to transient voiding problems. No operative intervention is required. S: symphysis pubis, BN: bladder neck, and H: hematoma.

1. If the sling is too close to the LSM complex (the distance < 3 mm) it is possible in the first seven postoperative days to loosen the implant by drawing on one of the sides preferably high with a Overholt clamp to avoid in particular suburethral damage of the band structure [21] (Figure 3).
2. In order to decide which side to draw on, it is necessary to evaluate the symmetry of the sling on the frontal and axial plane and then to evaluate the narrow or rather the urethra proximal side and then to loosen accordingly (Figure 4).
3. If the sling has been accidentally fixed with a vaginal suture, this presents a picture of a primary “tethered tape.” With physical exertion such as coughing the patient remains continent. However because the band is adhered to the vagina when the body posture is adjusted, for example, when standing up, this can result in the opening of the urethra and lead to subsequent urine loss. Appropriate plying of the sling from the adhesion will immediately solve the problem and also preserve the implant [22] (Figure 5).
4. If a hematoma is displacing the sling or compromising the urethra conservative treatment will be successful (Figure 6).

### 2.2. Late Complications (>7 Days)

In case of late complications occurring due to high faulty location of the sling, the vaginal part of the implant should be removed. A suburethral splitting alone is not sufficient.

**Figure 7:**
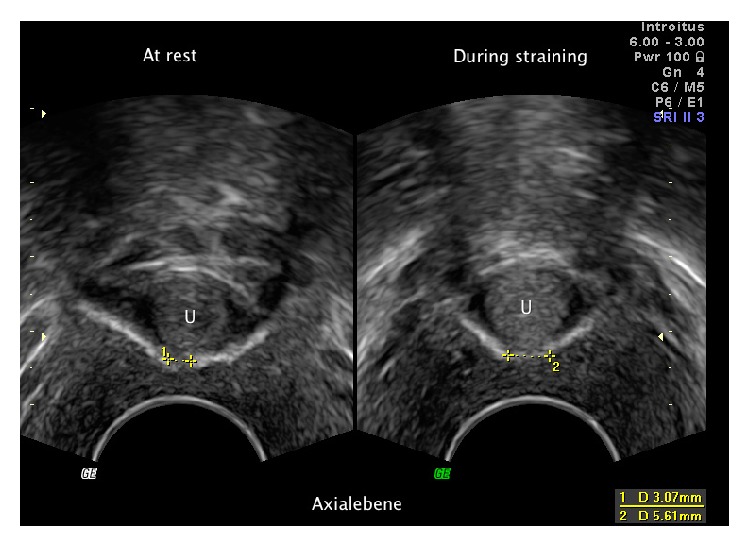
Pelvic floor ultrasound examination in axial plane. The sling was suburethrally split. Left: distance between both sling ends at rest. Right: during Valsalva manoeuvre.

In a narrow sling position, where the implant is close to the musculus sphincter urethrae externus, a suburethral sling splitting will loosen the sling but the continued fixed urethral muscle to the lateral sling ends can still irritate the urethra even at rest. OAB or draw on the urethra when the body is under strain can lead to urge or urine loss (Figure 7).

The primary suburethral split and laterally suppressed implant ends are difficult to locate even with the help of an ultrasound. The removal is extremely difficult.

**Figure 8:**
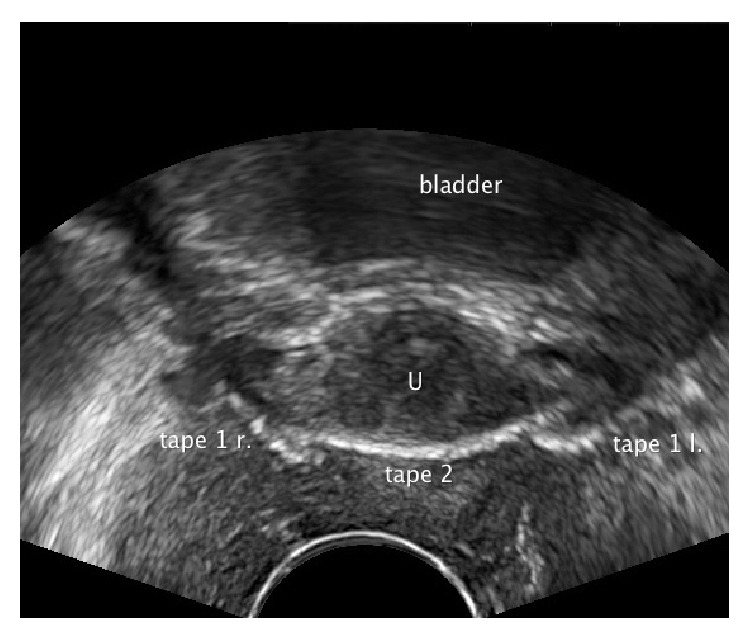
Pelvic floor ultrasound examination in axial plane. The sling was minimally removed vaginally. Both ends however lie still close to the urethra and disturb the second sling in its function: the so-called collision phenomenon. The patient is incontinent. Tape 1 r.: the right end of the first sling. Tape 1 l.: the left end of the first sling. Tape 2: the second sling.

In* low faulty position of the sling*, loosening at a later stage as mentioned above is not an option. In most cases a partial implant removal should be performed vaginally (*collision phenomenon*) (Figure 8).

Over 60% of patients have recurrent incontinence following a vaginal suburethral splitting. Removal of the sling ends at a later stage is almost impossible even with the support of interoperative PF-ultrasound examination. This is due to the fact that the implant can no longer be put under tension [23].

An exception is the repair of a low faulty positioned band, the so-called secondary “tethered tape.” The overtime adhered sling to the vagina can be made responsible for the recurrent incontinence. A vaginal adhesiolysis with or without gathering of the implant can improve the function of a vaginal sling and make continence possible [22].

## 3. Conclusions

Therapy failure after tension-free sling insertion is rare due to the method. Almost always it is possible to identify, with PF-ultrasound examination, the cause and also to solve the problem. With these investigation techniques and the described guidelines for the management of complications we would like to encourage the search for sling failure and in doing so, we actively provide the affected patients with an improvement of their problem.
